# Label-free NIR-SERS discrimination and detection of foodborne bacteria by in situ synthesis of Ag colloids

**DOI:** 10.1186/s12951-015-0106-4

**Published:** 2015-06-25

**Authors:** Longyan Chen, Nawfal Mungroo, Luciana Daikuara, Suresh Neethirajan

**Affiliations:** BioNano Laboratory, School of Engineering, University of Guelph, Guelph, ON N1G 2W1 12 Canada

**Keywords:** Surface-enhanced Raman scattering, Silver nanoparticles, Bacteria, Discrimination, In situ synthesis

## Abstract

**Background:**

Rapid detection and discrimination of bacteria for biomedical and food safety applications remain a considerable challenge. We report a label-free near infrared surface-enhanced Raman scattering (NIR-SERS) method for the discrimination of pathogenic bacteria from drinking water. The approach relies on the in situ synthesis of silver nanoparticles (Ag NPs) within the bacterial cell suspensions.

**Results:**

Pre-treatment of cells with Triton X-100 significantly improved the sensitivity of the assay. Using this method, we were able to discriminate several common pathogenic bacteria such as *Escherichia coli*, *Pseudomonas aeruginosa*, Methicillin-resistant *Staphylococcus aureus* (MRSA) and *Listeria spp*. A comparison of the SERS spectra allowed for the discrimination of two *Listeria* species, namely *L. monocytogenes* and *L. innocua*. We further report the application of the method to discriminate two MRSA strains from clinical isolates. The complete assay was completed in a span of 5 min.

**Conclusions:**

The proposed analytical method proves to be a rapid tool for selective and label-free identification of pathogenic bacterium. Pre-treatment of bacterial cells with Triton X-100 resulted in new features on the SERS spectra, allowing for a successful discrimination of common disease related bacteria including *E. coli*, *P. aeruginosa*, *Listeria* and MRSA. We also demonstrate that the spectral features obtained using in situ synthesis of nanoparticles could be could be used to differentiate two species of listeria. By using *L.innocua* as a model sample, we found the limit of detection of our assay to be 10^3^ CFU/mL. The method can selectively discriminate different bacterial species, and has a potential to be used in the development of point-of-care diagnostics with biomedical and food safety applications.

**Electronic supplementary material:**

The online version of this article (doi:10.1186/s12951-015-0106-4) contains supplementary material, which is available to authorized users.

## Background

Rapid detection and differentiation of pathogenic bacteria have become an increasingly important task in the pharmaceutical sector, medical and veterinary diagnostics, water and food safety, and food-processing industries. Conventional pathogen identification methods based on biochemical and microbiological tests requires a pure isolated bacterial culture and expensive equipment, which is laborious and time-consuming [1, 2]. Meanwhile the over usage of antibiotics in the clinical treatment of bacterial infections results in the emergence of “super bugs” bacterial species with undesirable antimicrobial resistance [3], which put additional cost to the public health budget. Advanced methods like polymerase chain reaction (PCR) and immunological detection generate results quicker than culture-based methods [4, 5]. However, they are often limited in regards to accuracy, specificity, speed, cost efficiency, versatility and sensitivity [6]. There is an urgent demand for a method that can deliver fast detection of pathogenic bacteria without the necessity of enrichment or culturing steps, and a detection limit in the 1–100 CFU per ml range [7, 8] leads to alternative approaches for rapid discrimination of pathogen.

Raman spectroscopy is becoming attractive for analysis of chemical and biological components [9, 10], even in a label-free modes. Raman spectroscopy has potential to detect a broad range of biological and chemical substances [11], and reveal the molecular composition of a sample at micrometer scale [12]. Raman signal of molecules can be dramatically enhanced by metallic nanoparticles (NPs) on the order of 10^4^–10^6^ using surface-enhanced Raman spectroscopy (SERS) via Surface Plasmon Resonance (SPR) [5, 13].

Surface-enhanced Raman spectroscopy techniques have been tested extensively for the detection, imaging, and discrimination of bacteria [11, 14–18]. Spectral signatures have been demonstrated to accurately distinguish gram-negative and gram-positive bacteria as the scattering intensity of gram positive bacteria has generally been observed to be higher than that of the gram negative bacteria across a range of wavelengths [19]. There are several approaches that have been employed for the detection of bacteria using SERS. Zhang and colleagues have reported the synthesis of magnetic–plasmonic Fe_3_O_4_–Au core–shell nanoparticles to concentrate bacterial cells by applying an external point magnetic field, as well as detection and identification of different bacteria using SERS [20]. More recently, Zhou et al. have reported a bio-sensing method for the detection of live bacteria in drinking water by coating with Ag nanoparticles [21] as well as detection of anthrax spores on nanosphere substrates [22]. A novel application employing SERS-active substrate composed of an array of Ag-nanoparticles imbedded in anodic aluminum oxide with nanochannels has been shown to exhibit a highly reproducible Raman signal enhancement factor due to the uniform narrow gaps between Ag-nanoparticles [23]. More complex nanohybrid systems developed by combining antibody-conjugated gold nanoparticles with single-walled carbon nanotubes have been used to detect multi drug resistant *Salmonella spp*. [24].

The predominant approach for the identification of bacteria by SERS is mixing as-made gold (Au NPs) and silver nanoparticles (Ag NPs) of various shapes or aggregates with bacterial cell suspension, as it is a label free method requiring minimal or no preparatory steps. Yet, it is very difficult to probe the fingerprints for various bacteria due to poor spectral reproducibility and selectivity through simple mixing process. Alternative method was directly producing NPs (in situ) in the bacterial cell suspension (through external-cell wall or internal-interior components modes), as reported by Efrima’s group [11, 18, 25]. This in situ approach ensures homogenous contact of constituents of the bacterial cells to NPs and hence gives an intense spectrum with unusual reproducibility and a better selectivity. However, the spectra obtained through an external mode are very similar among various bacterial species, which makes this mode unsuitable for bacterial discrimination from polymicrobial samples. Spectral similarities are attributed to flavin adenine dinucleotides (FAD), an important coenzyme known to be commonly present in the inner side of the bacterial cell wall [25]. Alternatively, the internal mode shows a distinct spectra with the absence of flavin spectral signature which is relatively weak due to a lesser degree of NPs aggregation [11]. Extraction of bacterial plasma from cell wall could possibly enhance the signal for the detection and discrimination. However, the treatment is rather time-consuming and not suitable for low concentration of bacterial cells. Smith-Palmer et al. [26] report that the SERS signal from FAD could be greatly reduced by excitation at near infrared (NIR) light source (785 nm) rather than the excitation at 514/532 nm, as reported by Efrima’s group [26]. The advantages of using NIR light source are that it allows for non-invasive probing of biological processes and discrimination of pathogenic bacteria in a low fluorescence background.

In this study, we report a label free NIR-SERS assay for discrimination and detection of bacteria in drinking water through in situ preparation of Ag NPs. We found that the selectivity of bacteria for discrimination could be enhanced greatly by the addition of cell membrane disruption reagent Triton X-100. By using the proposed method, we were able to discriminate two species of bacteria i.e., *Listeria* and within subtypes of bacterium such as Methicillin-resistant *Staphylococcus aureus* (MRSA) from clinical samples.

## Results and discussion

### NIR-SERS characterization of bacteria through in situ method

**Figure 1:**
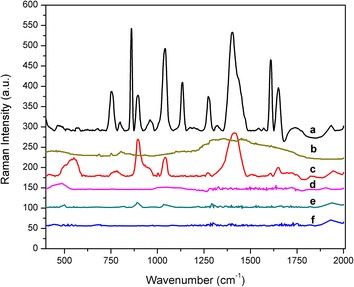
Averaged Raman spectra of (*a*) *E. coli* pre-treated with Triton X-100, (*b*) Triton X-100 only (with Ag NPs), (*c*) *E. coli* in external mode, (*d*) *E. coli* in internal mode, (*e*) Ag NPs only (without *E. coli* and Triton X-100) and (*f*) *E. coli* cells without Ag NPs.

Bacterium *Escherichia coli* was first used to demonstrate the applicability of NIR-SERS in situ assay. As shown in Figure 1, we could find distinguishable and sharp peaks from spectrum obtained by external mode (Figure 1c). In contrast, only a few weak bands (890 and 1,040 cm^−1^) were observed from the spectrum taken by internal mode (Figure 1d). In addition, the Raman spectrum for pure AgNPs did not show any identical bands (Figure 1e). Raman spectrum for bacterial samples without NPs shows a broad band around 1,100 cm^−1^ and another identical peak around 457 cm^−1^ (Figure 1d). This spectrum is not like those as reported elsewhere [27], where distinct bands could be found for *E. coli*. The lack of distinct spectral band for this measurement was attributed to the relatively low sensitivity of the Raman spectrometer. When the *E.coli* cells were incubated with Triton X-100, we observed a sharp increase in the peak intensities as well as the number of peaks in the spectrum (Figure 1a). Figure 1b shows the Raman spectrum of Triton X-100 in the presence of Ag NPs. No typical peaks were observed.

Fraction of bacterial cytoplasm could exhibit distinct Raman information for different bacterial cells [11]. However, as discussed above, the internal mode that collects the cytoplasmic information produces relatively lower intensity signal. On the other hand, it has been reported that Raman signals of biochemical information from cell interior could also be observed, by pre-treatment of cells with cell wall (or membrane) disrupting reagents such as antibiotics [23], ascorbic acid and Triton X-100 [25]. Triton X-100 is a detergent that can disrupt the integrity of the cell membranes, where the major components are lipids [28]. This results in exposure of inner components (such as proteins) from bacterial cells. Recently Reza Jalalirad [29] and Ma et al. [30] have shown selective permeabilization of proteins using Triton-X alone, without the presence of lysozyme.

We further examined the Raman spectra for *E. coli* by adding Triton X-100 (at a final concentration of 0.1%). We estimated that there might be some components released from cytoplasm due to the changes in the permeability of cell membrane. The result in Figure 1a shows the presence of several strong peaks when the bacterial cells were treated with Triton X-100. These new shifts include 753, 858, 1,134, 1,268, 1,608 and 1,656 cm^−1^. The tentative assignments of these shifts are listed in Additional file 1: Table S1, based on previous studies [16, 26, 31–33] (Additional file). Previous studies have reported that addition of Triton X-100 in human cells could increase the protein peaks in Raman spectra [34]. In our study, we observed the presence of new shifts namely, 753, 1,608 and 1,656 cm^−1^, which reflects the vibrations of tryptophan, tyrosine and amide I from the bacterial proteins, respectively. Other than Triton X-100, Tween 20, Brij 58, Lubrol WX, Brij 98, Brij 96 [35], chitosan [36] and thymol [37] could also be possibly used to improve the sensitivity of the assay.

### Characterization

**Figure 2:**
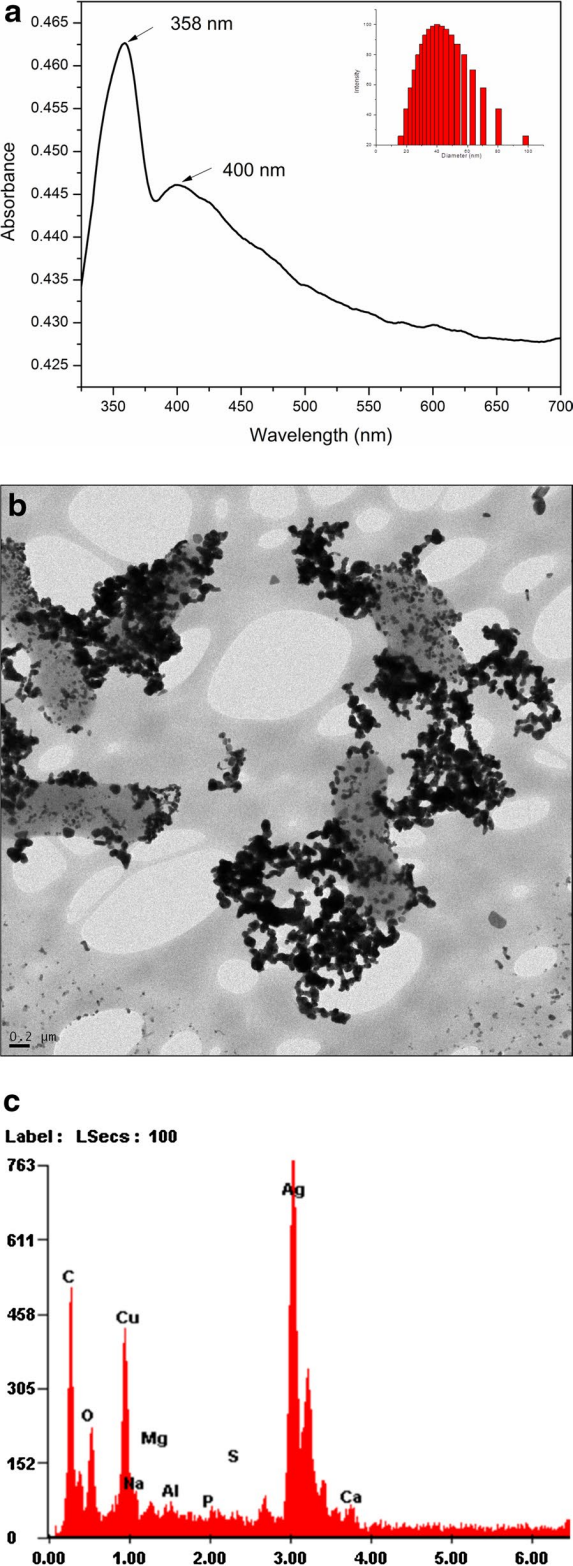
**a** UV–visible spectrum of Ag NPs prepared within bacteria mixture (external mode). **b** TEM image and **c** EDS analysis of the sample from external mode, respectively.

The as-made Ag NPs without *E. coli* appear as a yellow/greenish suspension. However in the presence of bacterial cells, the Ag NPs appear dark green in color. UV–vis spectrum indicates that there are two peaks at 358 and 400 nm for the Ag NPs made in the presence of bacterial cells (Figure 2a). The average diameter of Ag NPs measured by DLS is calculated to be 40 ± 8.4 nm (Figure 2a upright). Those two absorption peaks could correspond to the Ag NPs nuclei seeds and Ag NPs size growth. As Mie’s theory predicts only a single surface plasmon resonance (SPR) band for spherical Ag NPs, the observed two or more SPR bands are indicative of anisotropic morphology of our as-made Ag NPs (in the presence of bacterial cells) [38]. The wide band (shoulder) at 400–600 nm indicates the red-shift of spectrum due to the decrease in the inter-particle distance (formation of NPs aggregation) and/or an increase in the size of NPs. TEM image was further employed to demonstrate the formation of Ag NPs on the surface of bacteria by external mode (Figure 2b). A broader size distribution of NPs was observed in the TEM images. Figure 2b shows the formation of NPs aggregate on the surface of bacteria. EDS result further demonstrates that the NPs attributed to be silver (Figure 2c).

### NIR-SERS discrimination of bacteria

**Figure 3:**
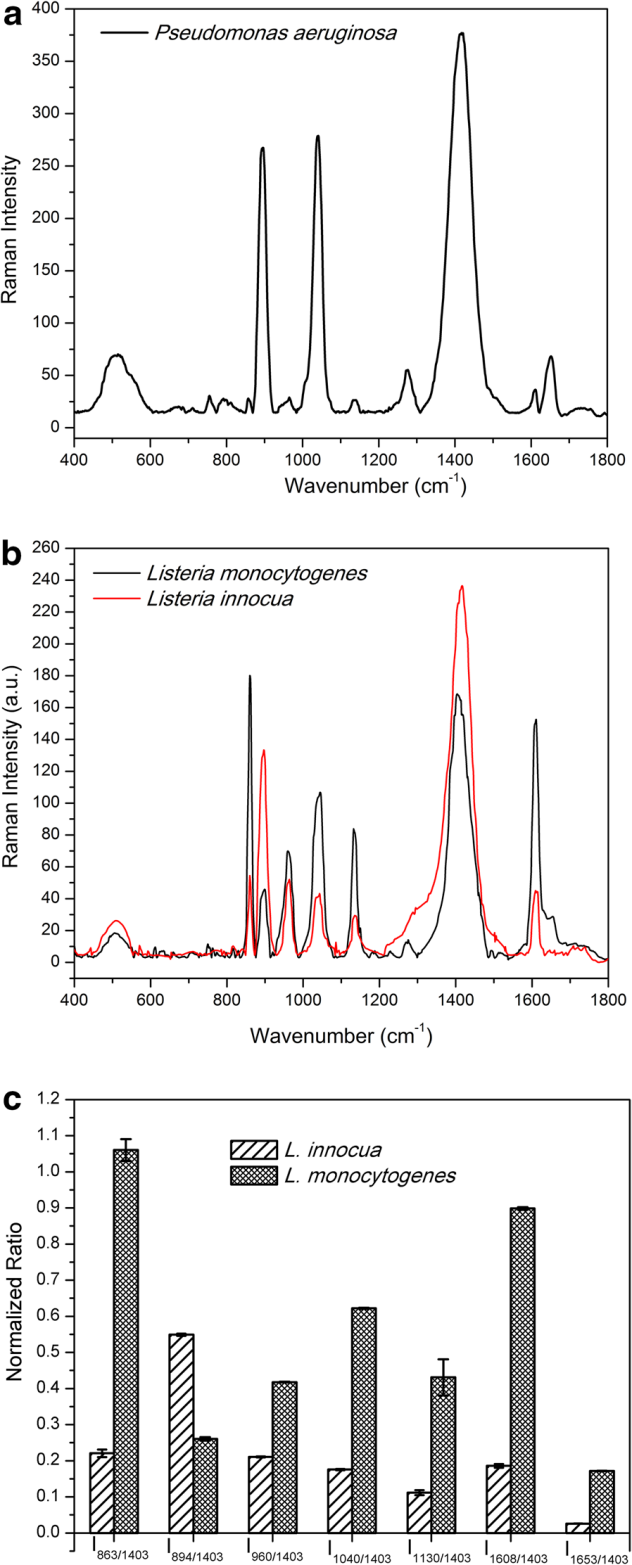
Averaged SERS spectra of **a** *P. aeruginosa* and **b** two species of Listeria (*L. monocytogenes* and *L. innocua*). **c** Normalized intensity ratio of significant peaks from two *Listeria spp.* to the band at 1,040 cm^−1^.

**Figure 4:**
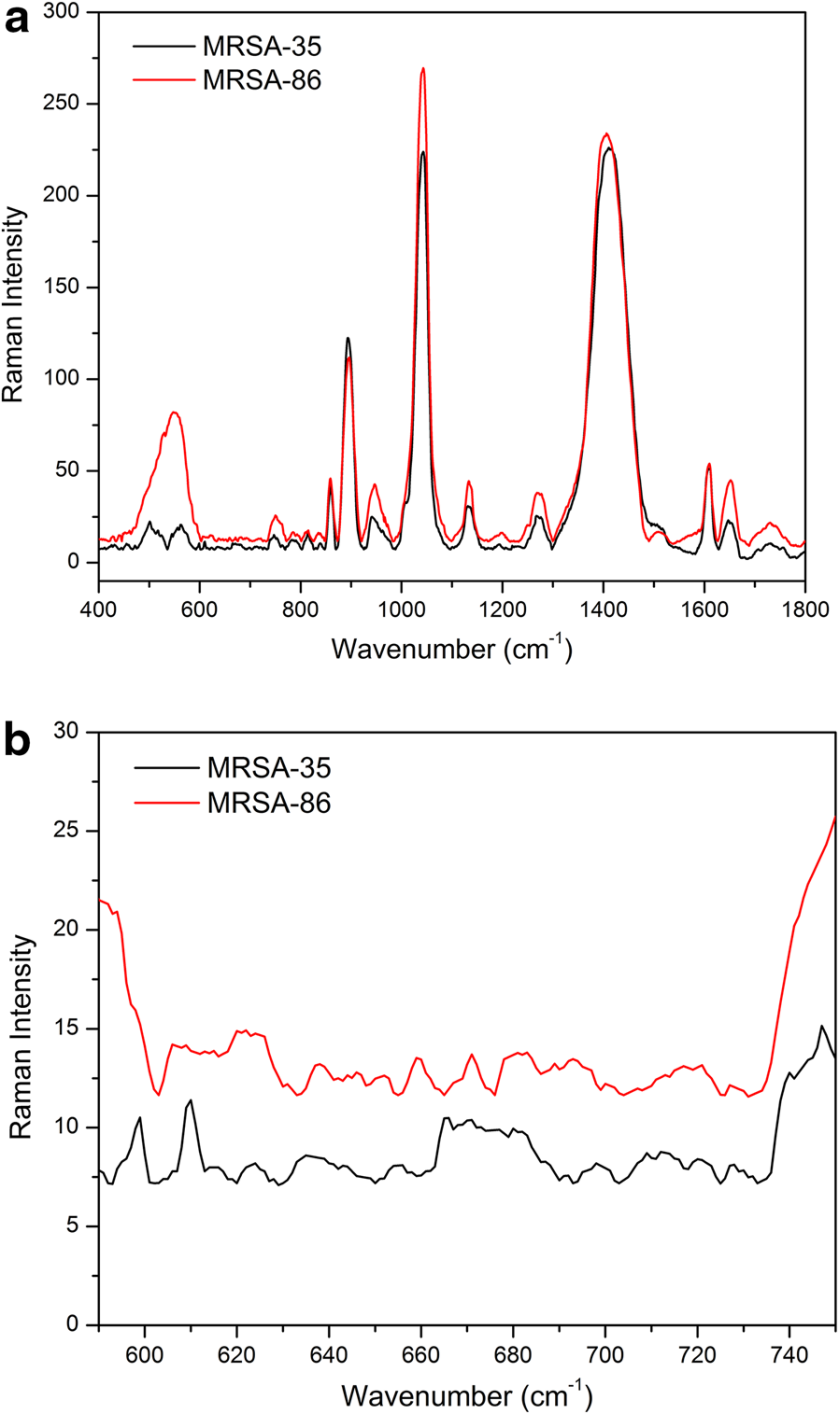
Averaged SERS spectra of **a** two strains of MRSA, **b** Zoom-in view of the spectra of two MRSA strains.

We then tested our modified SERS approach to characterize several common disease related bacterial species. Figures 3 and 4 show the spectral data for the bacterial species namely, *Pseudomonas aeruginosa*, *Listeria monocytogenes* and *Listeria innocua and* MRSA-86, MRSA-35. As can be seen in Figure 3, we were able to find the major Raman shifts for *P. aeruginosa* for positions at 893, 1,040 and 1,411 cm^−1^, which are attributed to be the vibrations from phosphodiester backbone, phenylalanine and COO– functional group respectively (details of assignments in Additional file 1: Table S1). It is observed that these three peaks are present in all the bacterial spectra studied in this report, though the relative intensities of the peaks vary among different bacteria. Figure 3b shows the SERS spectra for two species of *Listeria*. The positions of the major shifts are almost identical between the two species, including three peaks mentioned above and others (863, 946, 1,134, 1,237, 1,612 and 1,651 cm^−1^). Figure 3c shows the intensity ratio of significant peaks to the band at 1,040 cm^−1^. The band 1,040 cm^−1^ was selected as it has a strong intensity in all the bacterial species. The peak at 863/1,040 cm^−1^ for *L.monocytogenes* is significantly larger. Additionally, the band at 1,608 cm^−1^ is much stronger than the band observed for all other bacteria. In *L.innocua*, the largest ratio comes from the one at 894 cm^−1^ (Figure 3c). We were able to discriminate between the two species of *Listeria* using the relative ratios of these peaks. Furthermore, it could be observed that there is an obvious difference in the Raman frequency at position 1,658 cm^−1^ (Figure 3b), where the spectrum of *L.monocytogenes* shows a unique peak following the shoulder of the vibration band at 1,612 cm^−1^. Therefore, it could be concluded that we were able to efficiently and rapidly discriminate between the two Listeria species with the developed SERS method.

We further evaluated if our SERS approach could identify strains from the same bacterial species. As seen in Figure 4, the spectra from two MRSA strains exhibited highest peak intensity at the position 1,040 cm^−1^. The two MRSA strains show almost identical Raman spectra as observed in Figure 4a. Apart from the three major peaks, there are other peaks with medium intensity, including 863, 946, 1,134, 1,237, 1,612 and 1,651 cm^−1^ present in two MRSA strains. The band ascribed to be P–O–C binding of phospholipids at position 546 is present in MRSA-86. However, for MRSA-35, we found two peaks (unassigned) with medium intensity around the same location. As the differences and intensities of the bands are small, it is difficult to conclude that that they could be satisfactorily distinguish between the two different strains. The minor differences in peaks might also be due to disruption of cell wall by other factors such as mixing and centrifugation during the experiment. Albeit identical features in major peaks, we could find minor differences between the two strains in the peaks ranging from 600 to 700 cm^−1^. As can be seen in Figure 4b, a zoom-in view shows two small peaks (599 and 609 cm^−1^) and the appearance of a broad band around 670 cm^−1^ in MRSA-35. Meanwhile, in MRSA-86, there are peaks of spectral data with broadband at 610 cm^−1^ and another new peak at 659 cm^−1^. We were able to discriminate the two MRSA species based on these minor spectral differences.

**Figure 5:**
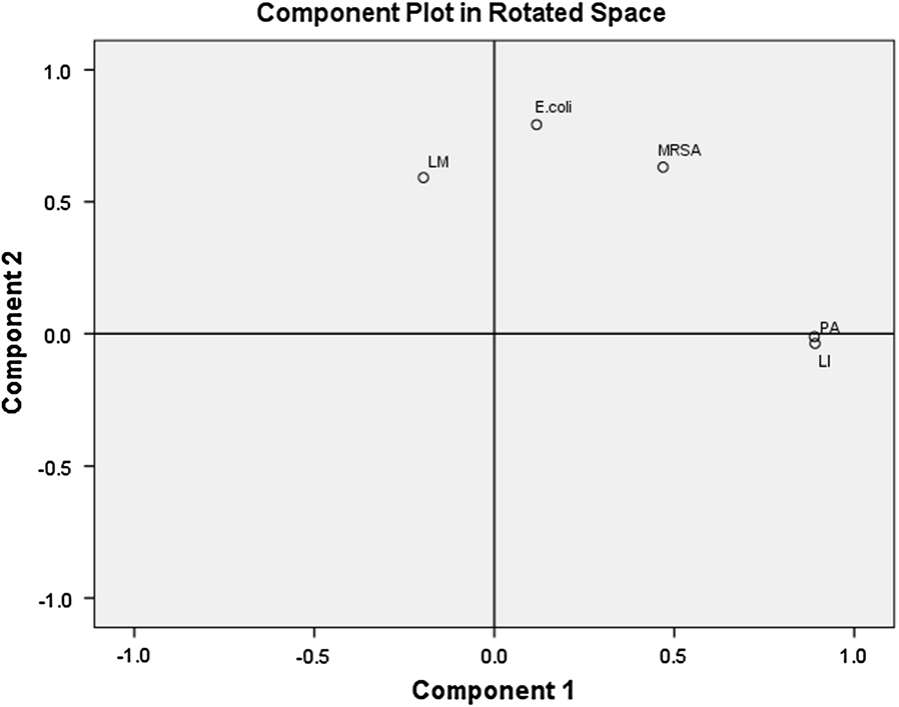
Ordination plot of the principal component analysis (PCA) applied to the RAMAN spectra of the five bacterial samples recorded at 25C. LM, *Listeria monocytogenes*; *E. coli,* 511 *Escherichia coli*; MRSA, *Methicillin Resistant Staphylococcus aureus*-*35*; PA, *Pseudomonas aeruginosa*; LI, *Listeria Innocua.*

Clustering of the bacterial spectrum samples can be seen in Figure 5. The results from the PCA analysis shows that each group of bacterial species separate out from each other allowing for discrimination between species. However, it can be noted that *L. innocua* showed a degree of overlap with *P. aeruginosa* (Figure 5). This could be due to the spectral interference from pigments pyocyanin and pyoverdine, which are released by the *Pseudomonas* species which was visually observed from the plates [39, 40].

For *P. aeruginosa,* biofilm embedded with carbohydrates, nuclei acids and proteins may also contribute to the spectral interference. A recent study revealed a significant increase in peak intensities attributed to carbohydrates and proteins during biofilm accumulation period [41]. It is possible that the Raman spectra of *P. aeruginosa* examined in our study are contributed by the biofilm components rather than the bacterial cell wall. As shown in Figure 3, apart from three relatively strong bands in the SERS spectrum of *P. aeruginosa,* which are attributed to nucleic acids (896 cm^−1^), carbohydrates (1,040 cm^−1^) and proteins (COO–, 1,411 cm^−1^), there are very few weaker peaks, suggesting that biofilm formation may directly contribute to the spectral interference for *P. aeruginosa*.

The results of this study provide a promising avenue for the development of a diagnostic platform for rapid discrimination, identification and classification of pathogenic microbial agents towards healthcare applications. Bacterial concentration may potentially obscure the relative peak intensity and the baseline intensity. However, modifying the surface of the substrates, and using microfluidics to avoid spectral interference in RAMAN spectra from mixed bacterial samples using the proposed in situ approach can achieve a uniform concentration of bacteria. The result of this study provides promising potential to develop a SERS based platform for field deployable, handheld point-of-care detection tool for rapid detection of pathogenic bacterial microorganisms including *Vibrio spp*. To achieve the above target, further miniaturization of the RAMAN spectrometer is a prerequisite. Challenges associated with the integration of the RAMAN spectrometer and the microfluidic device platform, and field based sample pre-processing protocols of the food matrix have to be considered towards the successful deployment of the NIR-SERS technique to discriminate and detect the foodborne pathogens.

### NIR-SERS detection of bacterium *Listeria innocua*

**Figure 6:**
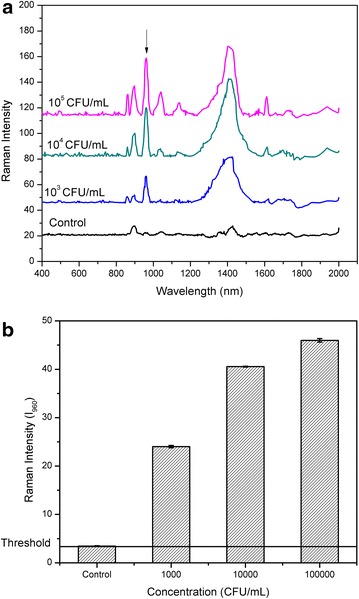
**a** SERS spectra of *L. innocua* at different concentrations (from 103 CFU/mL to 105 515 CFU/mL) and Nanopure™ water as a control. **b** Plot of the SERS peaks intensity at ∆v = 960 cm^−1^ (I960) against the bacterial concentration of *L. innocua.*

To investigate if the current assay mode could be used to determine the concentration of bacteria from SERS data, we collected the SERS spectra of *L.innocua* at different concentrations (from 10^3^ CFU/mL to 10^5^ CFU/mL) using Nanopure™ water as a control (Figure 6). This concentration window is considered to be a lethal dose of *Listeria. spp.* [42]. As discussed above, the peaks at ∆v = 860, 894 and 960 cm^−1^ were significantly stronger for *Listeria. sp*. Specifically, the intensity of the peak at 960 cm^−1^ increased as the bacterial concentration was increased. In addition, the peak corresponding to N–C stretching has been previously reported to be SERS peak for *Listeria sp*. by other groups [43]. Hence we believe that the peak at ∆v = 960 cm^−1^ could be used as signature peak used to estimate the concentration of *Listeria sp*. (*L. innocua* was used in the present study). In order to establish the limit of detection (LOD) for *L. innocua* samples, we plotted the SERS peak intensity at ∆v = 960 cm^−1^ (I960) with respect to the bacterial concentration (Figure 6b). At least nine spectra were measured for each concentration and analyzed by peak fitting. The mean and standard deviation of peak intensities are shown in Figure 6b. The value of average plus three times the standard deviation of the sterile DI water was set as the limit to distinguish a positive detection from negative detection. The lowest concentration of pure *L. innocua* detected by our assay was 10^3^ CFU/mL.

## Conclusions

We presented a label-free NIR-SERS approach for discrimination of bacteria in water through synthesis of Ag NPs within bacterial culture. We found that pre-treatment of Triton X-100 resulted in new features in SERS spectra, attributed to the inner components of the bacterial cell wall. Successful discrimination of common disease related bacteria including *E. coli, P. aeruginosa*, *Listeria* and MRSA were achieved. Furthermore, we were also able to identify two *Listeria* species (*L. monocytogenes* and *L. innocua*). Using the label-free NIR-SERS approach, we could also differentiate between two strains of MRSA isolated from clinical samples. The assay could be completed in less than 5 min. The assay requires very low volume of sample, which is a unique advantage. The developed assay can be used as a promising tool for selective discrimination of bacteria and will open new avenues for the development of point-of-care diagnostic devices with biomedical and food safety applications.

## Methods

### Chemicals and materials

Silver nitrate (Ag NO_3_), sodium borohydride (NaBH_4_), Triton X-100 and phosphate buffered saline (PBS) tablets were purchased from Sigma-Aldrich (Sigma-Aldrich, Oakville, Canada). Only Milli-Q water (18.2 mΩ cm) was used in this study. Bacterium *E. coli* (ATCC 25992) was purchased from ATCC. All other bacterial strains, including *L. innocua*, *L. monocytogenes* 1892, two strains of Methicillin-resistant *S. aureus* (MRSA) (USA100 defined as MRSA-35 and USA 300 defined as MRSA-86), and *P. aeruginosa* BK-76 used throughout this study, were isolated from clinical samples and received as gifts from the Canadian Research Institute for Food Safety (CRIFS) and the Ontario Veterinary College Hospital of the University of Guelph.

### Bacterial preparation

Bacterial cell suspensions were prepared from overnight cultures, grown overnight in 5 mL culture medium at 37°C and 150 rpm. For MRSA, the culture medium is brain heart infusion (BHI) broth (Oxoid Canada, Nepean, ON, Canada). For the other bacterial species, the culture medium was tryptone soy broth (TSB, Oxoid Canada, Nepean, ON, Canada). The bacterial cultures were then harvested and washed twice in water by centrifugation at 4,500*g* for 10 min at 4°C. Afterwards, the bacterial cells were resuspended in water for further analysis.

### SERS measurement

For SERS internal mode, harvested bacterial cells were resuspended in AgNO_3_ solution (1 M) and incubated for 5 min. The cells were then collected and washed twice in water by centrifugation at 4,500*g* for 10 min at 4°C. Afterwards, the cells were resuspended in NaBH_4_ solution (0.5 M) for further analysis. For SERS external mode, harvested bacterial cells were first resuspended in NaBH_4_ solution and incubated for 5 min. The cells were then collected by centrifugation and resuspended in AgNO_3_ solution for further analysis. In a typical experiment, the bacterial cells were treated with 0.1% Triton X-100 (v/v) for 5 min, prior to the assay. Then, 10 μL of treated bacterial suspension (~1 × 10^6^ CFU/mL) was mixed with 10 μL of NaBH_4_ (0.5 M). The mixture was then incubated for 3–5 min. Subsequently, 800 μL of AgNO_3_ (1 M) was pipetted into the mixture followed by vortexing.

Raman analysis was conducted by addition of 5 μL of the bacterial suspensions obtained from above modes onto the surface of glass cover slips (0.13 mm thickness, 15 mm diameter, Ted Pella Inc., Redding, CA, USA). SERS spectra were recorded on a benchtop Raman spectrometer (Sierra Snowy Range 785 series, Laramie, WY, USA) under excitation wavelength of 785 nm. The exposure time was 1 s and the number of accumulations for each measurement was 10. The spectral data were acquired over a Stokes Raman shift of 400–2,000 cm^−1^. For each study, three biological replicates were analyzed. To validate the results of the strain level discrimination, additional replicate was also analyzed.

### Statistical and PCA analysis

Statistical analysis was conducted using IBM SPSS Statistics 22 software. Principal component analysis was conducted using Matlab 2000b (The Mathworks, Inc., Natick, MA, USA) using the PLS toolbox (Eigenvector Research Inc., Wenatchee, WA, USA). Principal component analysis (PCA) was used to reduce the dimensionality of the multivariate data, and transform and identify the original set of variables into uncorrelated new variables through principal components. Upon plotting the principal components, the data from similar spectra were grouped as per the principal component scores. A total of 18 biological replicates for each bacterial species were used in acquiring the Raman spectra. Data analyses were performed on all the acquired Raman spectra for individual bacterial species. The SERS spectra were processed by taking the first derivative of the each spectrum and then were normalized to the unit vector length, followed by mean centering and auto-scaling prior to analysis of the data.

### Characterization

Transmission electron microscopy (TEM) images were acquired by Philips CM-10 equipment coupled with energy dispersive spectroscopy (EDS) operated at 120 kV. The UV–Vis absorption spectra were recorded by UV-3600 spectrophotometer (Shimadzu, Japan). The size distribution of Ag NPs in water was measured by dynamic light scattering with Zetasizer Nano ZS (Malvern Instrument Ltd. Worcestershire, UK), equipped with a glass cell.

## Additional file

Additional file 1:
**Table S1.** Tentative assignments^a^, and strength^b^ of peaks from SERS spectra of 5 bacteria [16, 26, 41–43].
