# Supplementary material for: Label-free NIR-SERS discrimination and detection of foodborne bacteria by in situ synthesis of Ag colloids
Source: J Nanobiotechnology. 2015 Jun 25;13:45. doi: 10.1186/s12951-015-0106-4 (PMC4479109; doi:10.1186/s12951-015-0106-4)
Supplement: Additional file 1: — Table S1. Tentative assignmentsa, and strengthb of peaks from SERS spectra of 5 bacteria [16, 26, 41–43]. [file 12951_2015_106_MOESM1_ESM.docx]

| *Pseudomonas aeruginosa* | *MRSA-35* | *MRSA-86* | *Listeria innocua* | *Listeria monocytogenes* | *Escherichia coli* | Assignment |
| --- | --- | --- | --- | --- | --- | --- |
| 517 (m) |  |  | 517 (m) | 517 (m) |  | S-S disulfide stretch in protein |
| 546 (m) | 556 (w) | 546 (s) |  |  |  | Carbohydrate |
| 659, 674 (w) |  | 659, 672 (w) |  |  |  | *δ* (guanine), ν (C-S) in cysteine. |
|  | 692 (w) |  |  |  | 681 (w) | (C-C) twist aromatic ring in Tyr |
| 757 (w) | 748 (w) | 749 (w) |  |  | 753 (m) | Trp |
| 797 (w) |  |  |  |  | 796 (w) | Cytosine, |
|  | 835 (w) | 835 (w) | 841(w) |  |  | *v (C-C) in 1,4 glycosidic link* |
| 857 (w) | 859 (m) | 858 (m) | 860 (m) | 860 (s) | 858 (s) | Ribose, *v* (COC) |
| 896 (s) | 894 (s) | 894 (s) | 894 (s) | 893 (m) | 892 (s) | Phosphodiester backbone, deoxyribose |
| 962 (w) | 942 (w) | 946 (m) | 960 (m) | 960 (m) |  | Phospholipids  N-C stretching |
| 1040 (s) | 1043 (s) | 1043 (s) | 1040 (m) | 1043 (s) | 1040 (s) | CC ring breathing ([polysaccharide](javascript:popupOBO('CHEBI:18154','C2AN16310A','http://www.ebi.ac.uk/chebi/searchId.do?chebiId=18154'))) |
| 1136 (w) | 1132 (w) | 1134 (m) | 1130 (w) | 1130 (m) | 1134 (s) | ν(COC), ring breathing |
|  | 1194 (w) | 1199 (w) |  |  | 1177 (w) | Amide III |
| 1275 (m) | 1272 (w) | 1273 (m) | 1291 (m, sh) | 1275 (w) | 1268 (m) | Amide I |
| 1411 (s) | 1410 (m) | 1408 (s) | 1403(s) | 1417 (s) | 1403 (s) | *v* (CO) symmetric COO- |
| 1509 (w, sh) | 1509 (w, sh) | 1511 (w) |  |  |  | Phe |
|  |  |  |  |  | 1544 (w) | v (CN) amide II, |
|  |  |  |  |  | 1567 (w) | N-Acetyl related, Amide II |
| 1609 (w) | 1610 (m) | 1608 (m) | 1608 (m) | 1608 (s) | 1608 (s) | Tyr |
| 1656 (m) | 1651 (w) | 1650 (m) |  | 1653 (m) | 1656 (m) | Amide I, protein |
|  | 1732 (w) | 1731 (w) | 1723 (w) | 1727(w) |  | ν(C=O) |
| ^a^Note: *δ*, deformation; *ν*, stretching; Phe, phenylalanine; Tyr, tyrosine, Trp, tryptophan.  ^b^Note: s, strong; m, medium; w, weak; sh, shoulder. | | | | | | |

Table 1 Tentative assignments^a^, and strength^b^ of peaks from SERS spectra of 5 bacteria [16, 26, 41-43].
